# Health-Related Quality of Life in Older Adults with Rheumatoid Arthritis: A Sex-Specific Case–Control Analysis of Physical and Mental Health Domains

**DOI:** 10.3390/jcm15051821

**Published:** 2026-02-27

**Authors:** Joan M. Nolla, Diego Benavent, Lidia Valencia-Muntalà, Laura Berbel-Arcobé, Paola Vidal-Montal, Judith Palacios-Olid, Montserrat Roig-Kim, Martí Aguilar-Coll, Javier Narváez, Carmen Gómez-Vaquero

**Affiliations:** Department of Rheumatology, IDIBELL-Hospital Universitari de Bellvitge, University of Barcelona, 08907 Barcelona, Spainjpalacios.hv@gencat.cat (J.P.-O.);

**Keywords:** rheumatoid arthritis, health-related quality of life, older adults, SF-12, physical and mental health, sex differences, patient-reported outcomes, case–control analysis

## Abstract

**Background:** The impact of rheumatoid arthritis (RA) on health-related quality of life (HRQoL) in older adults remains incompletely characterized, particularly regarding sex-specific patterns and the relative contribution of physical and mental health domains in later life. **Methods:** We conducted an observational cross-sectional case–control analysis including adults aged ≥65 years. A total of 180 patients with RA (65.6% women) were compared with 195 age- and sex-matched control subjects. HRQoL was assessed using the Short Form-12 (SF-12) questionnaire, generating Physical Component Summary (PCS) and Mental Component Summary (MCS) scores. Analyses were stratified by sex. Associations between HRQoL domains and clinical variables were examined using correlation analyses and sex-specific multivariable linear regression models. Effect sizes (Cohen’s d) were calculated to quantify the magnitude of between-group differences. **Results:** Women with RA showed significantly lower SF-12 physical (PCS) and mental (MCS) component scores than men with RA, indicating a greater impairment in overall HRQoL. Women also exhibited higher functional disability, as assessed by the Health Assessment Questionnaire (HAQ), and higher disease activity, as assessed by DAS28. In sex-stratified case–control comparisons, men with RA showed lower SF-12 PCS scores, while MCS scores were comparable to those of the age-matched male controls. In contrast, women with RA exhibited significantly lower PCS and MCS scores compared to age-matched female controls. In multivariable analyses, distinct sex-specific patterns were observed. In women with RA, HAQ emerged as the only independent determinant of PCS, whereas DAS28 was the sole independent determinant of MCS. In men with RA, PCS was independently associated with DAS28, whereas MCS was independently associated with HAQ. Effect size analyses indicated consistently small impairments in both physical and mental HRQoL domains in women, whereas in men, the impact was small and largely confined to the physical domain. **Conclusions:** In older adults with RA, HRQoL impairment is sex-dependent and domain-specific, with women experiencing a more pronounced and generalized reduction in both physical and mental health compared with men. Sex-specific differences in the relative contribution of disease activity and functional disability highlight the need for a differentiated interpretation of patient-reported outcomes in this population. A domain-specific and sex-aware assessment of HRQoL may enhance the clinical evaluation of older patients and provide a more comprehensive understanding of disease burden beyond inflammatory activity alone.

## 1. Introduction

Rheumatoid arthritis (RA) [1] is a chronic immune-mediated inflammatory disease characterized by persistent synovitis and systemic involvement. Although uncontrolled inflammation remains central to disease progression, major advances in early diagnosis, treat-to-target strategies and disease-modifying therapies have substantially improved inflammatory control and limited severe structural damage in many patients. Consequently, the contemporary burden of RA is no longer defined solely by objective measures of inflammation, but increasingly by its impact on daily functioning, well-being and overall health status [2]. In this context, health-related quality of life (HRQoL) [3] has become a key outcome, capturing the patient’s global experience of the disease and reflecting dimensions that extend beyond inflammatory activity alone.

Generic instruments such as the Short Form-36 (SF-36) [4] and its abbreviated version, the Short Form-12 (SF-12) [5], are widely used to assess HRQoL across a broad range of chronic conditions [6,7,8], and their reliability, validity and responsiveness are well established. Although not disease-specific, these instruments provide robust and standardized measures of physical and mental health, allowing meaningful comparisons between patient groups, reference populations and clinical settings. For these reasons, the SF-36 and SF-12 have been applied in RA research to characterize the overall impact of the disease and to contextualize patient-reported health status beyond disease-specific outcomes [9,10,11,12].

Despite the extensive literature on HRQoL in RA [13], several relevant gaps remain. Most published studies have focused on middle-aged cohorts, despite the growing proportion of older adults living with RA in contemporary clinical practice. Ageing [14,15] is associated with declining physiological reserve, increased systemic complexity and functional vulnerability, including a higher burden of comorbidities, all of which may substantially influence patient-reported health status. In this context, the relationship between inflammatory disease activity and perceived well-being may become less direct, leading to a potential misalignment between objective measures of disease control and HRQoL outcomes.

Sex-related differences further add complexity. RA is a sex-dependent disease, with well-established differences in prevalence, clinical expression and patient-reported outcomes between women and men [16,17]. Nevertheless, HRQoL is rarely analyzed in a sex-stratified manner [13], and potential differences in the relative impact of the disease on physical versus mental health domains remain insufficiently explored. This lack of stratified analysis may contribute to an incomplete understanding of how RA affects men and women differently, particularly in older age.

In this context, the present study aimed to characterize HRQoL in older adults with RA, with a specific focus on the physical and mental health domains assessed by the SF-12. Using a case–control design restricted to individuals aged 65 years or older, we compared patients with RA with age- and sex-matched control subjects and examined sex-specific patterns. In addition to identifying differences, we assessed the magnitude of impairment in physical and mental components to better delineate the relative impact of RA across domains in later life.

## 2. Materials and Methods

### 2.1. Study Design

This was an observational cross-sectional case–control study designed to evaluate HRQoL in older adults with RA.

### 2.2. Study Population

Patients with RA were recruited during routine follow-up visits at a tertiary university hospital rheumatology clinic. The present study represents a further investigation within the Bellvitge Rheumatoid Arthritis-Life Impact and Comorbidity Evaluation cohort (BELL-RA-LIFE), an ongoing single-center observational cohort that includes consecutive middle-aged and older adults with RA followed under routine clinical care.

The BELL-RA-LIFE cohort is specifically designed to characterize the global impact of RA across ageing, with a particular focus on comorbidity burden, nutritional and immune-related status, physical function, patient-reported outcomes, and HRQoL in real-world settings. Several complementary analyses derived from this cohort have previously addressed specific domains such as fatigue, sarcopenia risk, and nutritional vulnerability, each focusing on distinct outcomes and research questions.

For the purposes of the present study, HRQoL assessed using the SF-12 questionnaire was defined as the primary outcome. To specifically address the impact of RA in older age, analyses were restricted to participants aged 65 years or older.

### 2.3. Control Group

Control subjects were recruited from the same geographic area and were frequency-matched to patients by age and sex. Controls had no history of inflammatory rheumatic disease or other chronic inflammatory conditions. They were enrolled from both hospital-based and community sources, including accompanying relatives of outpatients, individuals with non-inflammatory musculoskeletal complaints, and subjects attending the hospital for non-musculoskeletal reasons.

Individuals with conditions associated with marked impairment of HRQoL or major systemic disease, including active malignancy or advanced heart, respiratory, liver, or kidney failure, were excluded. All participants underwent the same assessment of HRQoL and laboratory evaluation.

### 2.4. Demographic and Anthropometric Characteristics

Demographic and anthropometric variables were recorded in both patients with RA and control subjects, including age, sex, and body mass index (BMI). BMI was calculated as weight in kilograms divided by height in meters squared and categorized as underweight (<18.5 kg/m^2^), normal weight (18.5–24.9 kg/m^2^), overweight (25.0–29.9 kg/m^2^), or obese (≥30.0 kg/m^2^).

### 2.5. Clinical Assessment in RA

In patients with RA, disease-related variables were collected through clinical interview and review of medical records. These included disease duration and current pharmacological treatment.

Disease activity was evaluated using the Disease Activity Score in 28 joints (DAS28) [18], assessed at the time of inclusion by trained rheumatologists as part of routine clinical care. Higher DAS28 scores indicate higher levels of disease activity. Disease activity categories were defined according to established cut-off values.

Disability was assessed using the Health Assessment Questionnaire (HAQ) [19], a validated instrument that evaluates physical functioning based on difficulty in performing activities of daily living. Higher HAQ scores indicate greater disability.

Current treatment was recorded and classified as conventional synthetic disease-modifying antirheumatic drugs, biologic or targeted synthetic disease-modifying antirheumatic drugs, and glucocorticoid therapy, the latter recorded as a dichotomous variable.

### 2.6. Laboratory Assessment

Hemoglobin levels were recorded in both patients with RA and control subjects using the most recent available laboratory determination. In patients with RA, laboratory data corresponded to assessments performed within the three months preceding inclusion. In control subjects, values were obtained from the most recent routine blood analysis, with no relevant clinical changes expected between determinations.

### 2.7. Assessment of HRQoL

HRQoL was assessed in all participants using the SF-12 questionnaire version 1. The SF-12 is a validated generic instrument derived from the Short Form-36 that evaluates perceived health status across multiple chronic conditions and populations while minimizing respondent burden.

The SF-12 generates two standardized summary scores: the Physical Component Summary (PCS) and the Mental Component Summary (MCS). The PCS reflects physical functioning, role limitations due to physical health, pain interference, and general health perception, whereas the MCS captures mental health, emotional role functioning, vitality, and social functioning. PCS and MCS scores were calculated following the standard SF-12v1 scoring algorithm based on weighted item coefficients [5]. Scores were analyzed as continuous variables with higher scores indicating HRQoL and interpreted in comparison with the age- and sex-matched control group included in the study. The questionnaire was administered to both patients and control subjects, allowing direct comparison of physical and mental health domains between groups.

### 2.8. Statistical Analysis

Given the observational nature of the study, no formal a priori sample size calculation was performed. The sample size was determined by the total number of consecutive participants aged ≥65 years available within the BELL-RA-LIFE cohort during the study period, together with age- and sex-matched control subjects. To further contextualize the robustness of the findings, post hoc power analyses were conducted for the main comparisons. For the sex-stratified case–control analyses (patients with RA versus age- and sex-matched controls), the available sample size provided adequate power (>80%) to detect small-to-moderate differences in both PCS and MCS among women. In men, the study was adequately powered to detect moderate effects in physical health, whereas smaller effects, particularly in mental health outcomes, would require larger samples. In addition, for the comparisons between men and women within the RA group, the sample size was sufficient to detect moderate differences in SF-12 physical and mental component scores, supporting the robustness of the observed sex-related differences in HRQoL among patients with RA.

Continuous variables are presented as mean ± standard deviation or median (interquartile range). Categorical variables are expressed as absolute numbers and percentages.

Comparisons between patients with RA and control subjects were performed using Student’s *t*-test or the Mann–Whitney U test for continuous variables, and the chi-square test for categorical variables. The choice of statistical test was based on the distribution of the data, which was assessed using the Shapiro–Wilk test. Analyses were stratified by sex.

Within the RA group, bivariate associations between SF-12 Physical and Mental Component Summary scores and clinical or laboratory variables were assessed using correlation analyses (Pearson or Spearman, as appropriate).

Multivariable linear regression models were fitted separately in women and men with RA to identify independent determinants of PCS and MCS scores. Variables were selected for inclusion in the multivariable analyses based on their significant associations in the univariate analyses (age, body mass index, hemoglobin level, HAQ, ESR, CRP, and DAS28). Effect sizes were estimated using r derived from the Mann–Whitney U test to quantify the magnitude of differences between patients with RA and control subjects for the physical and mental components of the SF-12, allowing comparison across domains and between sexes.

A two-sided *p*-value < 0.05 was considered statistically significant. Statistical analyses were performed using IBM SPSS Statistics for Windows, version 22.0 (IBM Corp., Armonk, NY, USA).

### 2.9. Ethical Considerations

The study was conducted in accordance with the Declaration of Helsinki and was approved by the Ethics Committee of Hospital Universitari de Bellvitge (protocol PR057/20). All participants provided written informed consent prior to inclusion.

## 3. Results

Baseline sociodemographic and clinical characteristics of patients with RA are summarized in Table 1. Within the cohort, 65.6% (n = 118) were women and 34.4% (n = 62) were men.

Men were older than women [76.0 (71.0–81.0) vs. 71.0 (68.0–77.0) years; *p* < 0.01] but had a shorter disease duration [11.0 (5.5–17.5) vs. 16.5 (11.0–24.0) years; *p* < 0.01]. Women showed slightly higher disease activity, as assessed by DAS28 [2.82 (2.10–3.57) vs. 2.25 (1.55–3.04); *p* < 0.05], and greater functional disability, as assessed by HAQ [0.50 (0.12–1.00) vs. 0.12 (0.00–0.62); *p* < 0.01].

Women with RA reported poorer HRQoL in both the physical [PCS: 35.0 (30.0–43.0) vs. 44.5 (36.0–51.0); *p* < 0.001] and mental [MCS: 46.0 (36.0–55.0) vs. 52.5 (46.5–58.0); *p* < 0.01] components of the SF-12 compared with men with RA.

In men with RA, SF-12 PCS scores were significantly lower than those of age- and sex-matched control subjects [44.5 (36.0–51.0) vs. 49.7 (38.5–53.8); *p* < 0.05], whereas no significant differences were observed in MCS scores. In contrast, women with RA exhibited significantly lower HRQoL compared with age- and sex-matched controls. Both the PCS score [35.0 (30.0–43.0) vs. 52.6 (32.7–41.5); *p* < 0.01] and the MCS score [46.0 (36.0–55.0) vs. 52.0 (41.2–58.1); *p* < 0.01] were significantly reduced in women with RA.

As shown in Table 2, in men with RA, PCS was significantly associated with functional disability measured by HAQ (r = −0.354; *p* < 0.01), and disease activity assessed by DAS28 (r = −0.379; *p* < 0.01). MCS was significantly associated with functional disability (HAQ) (r = −0.273; *p* < 0.05).

In women with RA, PCS was significantly associated with age (r = −0.190; *p* < 0.01), BMI (r = −0.163; *p* < 0.05), hemoglobin levels (r = 0.240; *p* < 0.001), HAQ (r = −0.448; *p* < 0.001), ESR (r = −0.286; *p* < 0.05), and DAS28 (r = −0.390; *p* < 0.01). MCS was significantly associated with inflammatory markers and disease activity, including CRP levels (r = −0.346; *p* < 0.01) and DAS28 (r = −0.246; *p* < 0.05).

In multivariable linear regression analyses, distinct sex-specific patterns emerged. In women with RA, HAQ emerged as the only independent determinant of PCS (adjusted R^2^ = 0.448), whereas DAS28 was the sole independent determinant of MCS (adjusted R^2^ = 0.101). In men with RA, PCS was independently associated with DAS28 (adjusted R^2^ = 0.132), whereas MCS was independently associated with HAQ (adjusted R^2^ = 0.061). A combined model including DAS28 and HAQ explained a greater proportion of variance in PCS in men (adjusted R^2^ = 0.229).

Effect size analyses are presented in Table 3. These estimates indicate a more pronounced impact of RA on both physical and mental HRQoL domains in women, whereas in men the impact was smaller and largely confined to the physical domain.

## 4. Discussion

In this case–control study of adults aged 65 years or older, we characterized HRQoL in RA through sex-stratified analyses of the physical and mental components of the SF-12. By comparing patients with age- and sex-matched control subjects and examining these domains separately, we provide a focused assessment of the impact of RA in later life. Our findings show that this impact is heterogeneous and differs by sex and by health domain, highlighting the need for a more nuanced interpretation of patient-reported outcomes in older adults.

Previous studies have consistently reported poorer HRQoL in women with RA than in men; however, evidence based specifically on the SF-12 remains limited. In a large cross-sectional cohort, Linde et al. [12] identified female sex as an independent determinant of worse MCS, with no association observed for PCS. Importantly, analyses were not sex-stratified, the study population was not restricted to older adults, and comparisons with matched control subjects were not performed.

A recent systematic review [13] further highlights these gaps, showing that although sex is frequently included as a covariate, sex-stratified analyses are uncommon, physical and mental components are rarely examined as distinct domains, and most data derive from mixed-age observational cohorts without matched control populations. In this context, our findings extend previous work by demonstrating sex-specific differences across both physical and mental domains of HRQoL in an exclusively older population, using a matched case–control design and domain-specific analyses.

Separate evaluation of physical and mental domains provided additional insight. In our study, physical health (PCS) was more closely related to inflammatory disease activity, whereas mental health (MCS) showed a weaker and more heterogeneous association with inflammatory markers, particularly in men. Moreover, distinct sex-specific patterns emerged: in women, functional disability was the main determinant of physical health, while disease activity independently influenced mental health; in contrast, in men, disease activity was associated with physical health, whereas functional disability was the primary determinant of mental health. These findings suggest that treating HRQoL as a single composite outcome may obscure clinically meaningful patterns in older adults and support the value of domain-specific analyses in RA.

The sex-specific determinants of physical and mental HRQoL observed in this study may reflect differences in how inflammatory activity and functional limitation are perceived and integrated into overall health in later life. In women, functional disability may act as an integrative marker of cumulative disease burden, capturing not only joint impairment but also pain, fatigue, and comorbidity, thereby exerting a stronger influence on physical HRQoL. Conversely, mental HRQoL in women may be more sensitive to ongoing inflammatory activity and its symptomatic expression, including pain and stiffness, which can disproportionately affect emotional well-being. In men, physical HRQoL may remain more closely aligned with current inflammatory status, whereas mental HRQoL may be more strongly influenced by loss of functional autonomy as reflected by disability. Although these interpretations are necessarily speculative, they suggest that the pathways linking inflammation, disability, and perceived health are not uniform across sexes in older adults with RA.

These patterns should be interpreted in the context of ageing-related changes in RA. Ageing [15,20] is associated with increased comorbidity, reduced physiological reserve and greater systemic complexity, which may weaken the link between inflammatory control and perceived well-being in later life. Consistent with concepts of immune ageing and inflammaging [14,15], age-related immune dysregulation and persistent low-grade inflammation may contribute to a more complex and less linear relationship between disease activity and patient-reported outcomes, with differential effects on physical and mental health domains and persistent sex-specific differences [14,15,21].

While statistical testing determines whether between-group differences are unlikely to be due to chance, it does not capture their magnitude. We therefore examined effect sizes for the physical and mental components of the SF-12, providing a standardized estimate of HRQoL impairment that facilitates comparisons across domains, sexes and sample sizes. From a clinical perspective, even small effect sizes may represent clinically meaningful differences in older adults, in whom limited functional reserve may amplify the impact of relatively modest changes in physical or mental health domains. In older populations, where clinical heterogeneity is high, effect sizes are particularly informative, as non-significant findings may reflect limited statistical power rather than absence of impact. In this context, our results show clear sex- and domain-specific patterns: among older adults with RA, women exhibit small but clinically meaningful impairments across both physical and mental domains, whereas in men the impact is smaller and largely confined to physical health. These findings highlight the value of domain-specific and sex-stratified assessment of HRQoL in older patients with RA, as conventional measures of inflammatory control may not fully reflect perceived health status, particularly with respect to mental well-being.

The sex-specific patterns observed in the multivariable analyses warrant careful interpretation. In men, the associations appear clinically coherent: disease activity (DAS28) was independently associated with physical health (PCS), whereas functional disability (HAQ) was associated with mental health (MCS). This configuration is consistent with the conceptual proximity between inflammatory burden and physical functioning, and between loss of autonomy and psychological well-being. In women, a different pattern was observed. HAQ was independently associated with PCS, which may reflect the partial conceptual overlap between functional disability and perceived physical health, as both instruments capture related aspects of daily functioning. More notable was the independent association between DAS28 and MCS. Although causal inferences cannot be drawn, this finding raises the possibility that, in older women, inflammatory activity and its symptomatic expression—potentially including dimensions not fully captured by DAS28 or HAQ, such as fatigue, pain amplification, or sleep disturbance—may be more closely aligned with perceived mental well-being. Differences in symptom perception, reporting patterns, and coping strategies between sexes could also contribute to this configuration. In addition, contextual factors, such as caregiving roles that remain more prevalent among older women, may be relevant when interpreting the observed associations, although these variables were not directly measured in the present study. These considerations are hypothesis-generating and should be explored in future research incorporating direct measures of psychosocial and contextual factors.

This study has several strengths. The case–control design with age- and sex-matched control subjects allows a more robust contextualization of HRQoL in older adults with RA than studies relying on normative reference data. The exclusive focus on individuals aged 65 years or older, together with the separate analysis of physical and mental health domains and sex-stratified analyses, provides novel and clinically relevant insights into the heterogeneous impact of RA in later life.

Some limitations should also be acknowledged. The cross-sectional design precludes causal inferences regarding the observed associations. The study was conducted at a single center, which may limit generalizability, and unmeasured factors such as psychosocial variables (including depression or socioeconomic status) could have influenced patient-reported outcomes. Nevertheless, our use of validated instruments and inclusion of a well-characterized cohort support the robustness of the findings. Finally, the lower number of men relative to women may have reduced the ability to detect very small differences in some sex-stratified analyses, especially for mental health domains; however, the sample size was sufficient to detect moderate and clinically relevant effects.

## 5. Conclusions

Among older adults with RA, the impact of the disease on HRQoL is heterogeneous and varies by sex and across physical and mental domains. Our findings indicate that functional disability and inflammatory disease activity contribute differently to physical and mental health depending on sex and that disease activity alone does not fully capture the burden of RA in later life, particularly with respect to mental well-being. A domain-specific and sex-aware interpretation of patient-reported outcomes may therefore improve the clinical assessment of older patients and support a more individualized approach to disease management beyond conventional measures of inflammatory control.

## Figures and Tables

**Table 1:** Characteristics of the 180 RA patients, stratified by sex.

	Men (n: 62)	Women (n: 118)	*p*-Value
Age (years)	76.0 (71.0–81.0)	71.0 (68.0–77.0)	<0.01
BMI (kg/m^2^) Underweight (n, %) Normal range (n, %) Overweight (n, %) Obese (n, %)	27.9 (24.8–29.4) 1 (1.6%) 16 (25.8%) 35 (56.5%) 10 (16.1%)	26.9 (23.7–30.0) 0 40 (33.9%) 47 (39.8%) 31 (26.3%)	ns ns
Smoking * Never (n, %) Former (n, %) Current (n, %)	54 (85.7%) 1 (1.2%) 7/(13.1%)	106 (87.2%) 2 (1.1%) 9/117 (11.7%)	ns
Physical activity ** None (n, %) Sporadic (n, %) Regular with low intensity (n, %) Regular with high intensity (n, %)	21 (33.9%) 10 (16.1%) 27 (43.5%) 4 (6.5%)	58 (50.0%) 23 (19.8) 35 (30.2%) 0	<0.01
Hemoglobin (g/dL) Serum albumin (g/L)	13.9 ± 1.5 43 (40–45)	13.3 ± 1.2 44 (42–46)	<0.01 <0.01
Disease duration (years)	11.0 (5.5–17.5)	16.5 (11.0–24.0)	<0.01
RF + (n, %) RF titer (UI/L)	34/58 (60.2%) 51.9 (16.2–133.9)	76/105 (73%) 58.4 (26.0–190.9)	ns ns
ACPA + (n, %) ACPA titer (U/L)	33/59 (62.6%) 173 (26–471)	70/107 (69%) 175 (71–365)	ns ns
Current medication Glucocorticoids (n, %) cDMARDs (n, %) bDMARDs (n, %) Jak inhibitors (n, %)	35 (54.7%) 54 (86.9%) 12 (23.8%) -	53 (46.5%) 105 (90%) 40 (36%) -	ns ns <0.05
ESR (mm/h)	13.5 (6.0–33.0)	16.5 (8.5–31.5)	ns
CRP (mg/dL)	4.0 (1.6–9.8)	2.4 (1.6–6.6)	<0.05
DAS28 Remission (n, %) LDA (n, %) MDA (n, %) HDA (n, %)	2.25 (1.55–3.04) 36 (58.1%) 11 (17.7%) 12 (19.4%) 3 (4.8%)	2.82 (2.10–3.57) 50 (42.4%) 29 (24.6%) 36 (30.5%) 3 (2.5%)	<0.05 ns
HAQ	0.12 (0.00–0.62)	0.50 (0.12–1.00)	<0.01
SF-12 PCS MCS	44.5 (36.0–51.0) 52.5 (46.5–58.0)	35.0 (30.0–43.0) 46.0 (36.0–55.0)	<0.001 <0.01

BMI, body mass index; RF, rheumatoid factor; ACPA, anti-citrullinated peptides antibodies; ESR, erythrocyte sedimentation rate; CRP, C-reactive protein; cDMARDs, conventional disease-modifying antirheumatic drugs; bDMARDs, biological disease-modifying antirheumatic drugs; DAS28, Disease Activity Score 28; LDA, low disease activity; MDA, moderate disease activity; HDA, high disease activity; HAQ, Health Assessment Questionnaire; SF-12, Short Form Health Survey-12.; PCS, Physical Component Summary; MCS, Mental Component Summary. * data for two women were missing. ** data for one woman were missing. “ns” indicates non-significant differences.

**Table 2:** Comparison of demographic and clinical characteristics between male and female patients with rheumatoid arthritis (RA) and between each sex-specific RA group and their respective age-matched controls.

	Men			Women		
	Patients (n: 62)	Controls (n: 75)	*p*	Patients (n: 118)	Controls (n: 120)	*p*
Age (years)	76.0 (71.0–81.0)	75.0 (70.0–79.5)	ns	71.0 (68.0–77.0)	73.0 (68.0–77.5)	ns
BMI (kg/m^2^) * Underweight (n, %) Normal range (n, %) Overweight (n, %) Obese (n, %)	27.9 (24.8–29.4) 1 (1.6%) 16 (25.8%) 35 (56.5%) 10 (16.1%)	26.8 (24.5–29.6) 0 24 (32.0%) 34 (45.3%) 17 (22.7%)	ns ns	26.9 (23.7–30.0) 0 40 (33.9%) 47 (39.8%) 31 (26.3%)	27.9 (25.4–31.3) 3 (2.6%) 20 (17.1%) 51 (43.5%) 43 (36.8%)	ns <0.01
Hemoglobin (g/dL)	13.9 ± 1.5	14.4 ± 1.7	ns	13.3 ± 1.2	13.6 ± 1.2	<0.05
SF-12 PCS MCS	44.5 (36.0–51.0) 52.5 (46.5–58.0)	49.7 (38.5–53.8) 52.8 (47.7–58.8)	<0.05 ns	35.0 (30.0–43.0) 46.0 (36.0–55.0)	52.6 (32.7–41.5) 52.0 (41.2–58.1)	<0.01 <0.01

BMI, body mass index; SF-12, Short Form Health Survey-12; PCS, Physical Component Summary; MCS, Mental Component Summary. * BMI data were missing for three women in the control group.

**Table 3:** Differences in SF-12 Physical and Mental Component Scores and Effect Sizes.

SF-12	Men			Women		
	Patients (n: 62)	Controls (n: 75)	Effect Size (r)	Patients (n: 118)	Controls (n: 120)	Effect Size (r)
PCS	44.5 (36.0–51.0)	49.7 (38.5–53.8)	0.18	35.0 (30.0–43.0)	52.6 (32.7–41.5)	0.22
MCS	52.5 (46.5–58.0)	52.8 (47.7–58.8)	0.02	46.0 (36.0–55.0)	52.0 (41.2–58.1)	0.199

SF-12: Short Form Health Survey-12; PCS: Physical Component Summary; MCS: Mental Component Summary.

## Data Availability

The datasets used and analyzed during the current study are available from the corresponding author upon reasonable request.
